# A Retrospective Study on Bacteriology, Clinicopathologic and Radiographic Features in 28 Cats Diagnosed with Pyothorax

**DOI:** 10.3390/ani11082286

**Published:** 2021-08-03

**Authors:** Juin Jia Sim, Seng Fong Lau, Sharina Omar, Malaika Watanabe, Muhammad Waseem Aslam

**Affiliations:** 1 Department of Veterinary Clinical Studies, Faculty of Veterinary Medicine, University Putra Malaysia, Serdang 43400, Malaysia; maraika@upm.edu.my (M.W.); dr.waseemaslam@gmail.com (M.W.A.); 2 Laboratory of Cancer Research UPM-MAKNA (CANRES), Institute of Bioscience, University Putra Malaysia, Serdang 43400, Malaysia; 3 Department of Veterinary Pathology and Microbiology, Faculty of Veterinary Medicine, University Putra Malaysia, Serdang 43400, Malaysia; sharina@upm.edu.my

**Keywords:** feline, pyothorax, radiograph, bacteria, antimicrobial resistance

## Abstract

**Simple Summary:**

The cat is a popular pet in Malaysia. Pyothorax is one of the most common respiratory conditions of cats in Malaysia. The etiological agents, particularly bacteria, of local feline pyothorax cases have never been studied. Therefore, in the present study we aimed to identify the bacteria involved in feline pyothorax cases, detect the antimicrobial susceptibility of the bacteria involved and study the radiographic findings related to these cases. Clinical records of cats diagnosed with pyothorax between 2013 and 2020 were analysed. The cats were presented with respiratory signs. Blood results showed significant inflammatory patterns and radiographs revealed different findings for each cat. The common bacteria and the antimicrobial susceptibility of the bacteria were determined in this study. Reporting the most common bacteria involved will give clinicians useful information that will further help in the selection of appropriate treatment and help clinicians understand the pathogenesis of pyothorax in the Malaysian cat population.

**Abstract:**

This retrospective study aimed to determine the etiological, clinicopathological, and radiographic features and outcome of feline pyothorax cases. Medical records from twenty-eight cats with pyothorax aged from 4 months to 10 years (median 10 months) diagnosed between 2013 and 2020 were reviewed. Dyspnoea (75.0%), abnormal lung sounds (75.0%) and open-mouth breathing (64.3%) were the predominant respiratory signs. Leucocytosis (61.5%), particularly monocytosis (68.0%), and hyperglobulinaemia (65.4%) were among the most prominent findings in blood analysis. Bilateral pleural effusion was found in 67.9% of the thoracic radiographs. A total of 47.4% of the cytological samples revealed the presence of bacteria, while all had positive bacterial growth. *Pasteurella multocida*, *E. coli*, *Streptococcus* spp., and *Staphylococcus* spp. were the predominant aerobic bacteria isolated from pleural effusion samples. A chest tube was placed in 64.3% of the cats and 66.7% of cats with chest tubes survived. In total, 46.4% of cats with pyothorax recovered. Amoxicillin–clavulanate was the antimicrobial of choice against aerobic bacteria found in this study and should be given in combination with antimicrobials that cover anaerobic bacteria. Chest tube placement is crucial for treatment success. Cytological results and bacterial culture may not be consistent; thus, bacterial culture should be performed for every case.

## 1. Introduction

Cats are the most common pets in Malaysian households and live closely with humans. Most Malaysian feline owners own an average of two to three cats, with some having more than 10 cats per household, and they are often managed as semi-roamers [1,2]. Access to the outdoor environment predisposes cats to exposure to infectious agents due to increased encounters with other sick or carrier cats [3]. Moreover, a recent survey of a single Malaysian state revealed that only 30% of the pet population, including cats, were vaccinated against feline herpesvirus-1, feline calicivirus and *Chlamydia psittaci* [4].

Pyothorax is one of the most commonly seen respiratory conditions among cats in Malaysia. It is defined as the accumulation of septic purulent exudate within the pleural space [5]. More than 80% of feline pyothorax is caused by mixed anaerobic bacterial infections [6]. Feline pyothorax is associated with polymicrobial infection consisting of obligate anaerobes (such as *Clostridium* spp., *Fusobacterium* spp., *Bacteroides* spp.) and/or facultative aerobic bacteria (which include *Pasteurella* spp. and *Actinomyces* spp.) [7,8]. Less than 20% of cases are caused by non-oropharyngeal bacteria [9,10], such as *Escherichia coli*, *Salmonella* spp., *Klebsiella* spp., *Pseudomonas* spp., and *Nocardia* spp. [7], whereas *Mycoplasma* spp. is a potential cause of pyothorax in kittens [11] and immunosuppressed adults [12].

The underlying etiopathogenesis in feline pyothorax cases is often unclear [13]. The aetiology of most of the cases remained unidentified and post mortem examination was required to identify the etiopathogenesis of these cases [8]. A study of 50 cases of canine and feline pyothorax in the United Kingdom and Ireland reported the underlying cause of pyothorax in only 18% of the cases [14]. In a retrospective study of 27 cats in Australia, the etiopathogenesis of pleural space infection was identified in 67% of cats and parapneumonic pyothorax was the greatest hypothesised etiopathogenesis in 15 out of 27 (56%) cats [13].

Haematogenous or lymphatic spread, direct inoculation through a penetrating bite wound, parasitic migration, parapneumonic spread and foreign-body migration are suggested routes of feline pyothorax infection [7,8,15]. Viral upper respiratory infections have been described to play a role in pleuropneumonia or pyothorax in cats, horses and humans by temporarily damaging mucociliary clearance function [16,17,18,19]. Commensals of the upper respiratory tract and oral cavity have been identified as the most common causative agents in pyothorax [5].

Clinically, pyothorax cats often manifest with dyspnoea, abnormal lung sounds or muffled heart sounds upon auscultation and other non-specific signs such as inappetence and lethargy. Radiography should be the first-line diagnostic imaging modality for feline pyothorax cases [5]. Radiographic signs such as lung lobe retraction, the enhancement of lobar edges and interlobar fissures are classical signs of pleural effusion [13]. Pleural effusion often has the characteristic of turbid to opaque septic exudates of cream or pale-yellow colour; however, it can also be pink, green or red-tinged. Cytologically, a high nucleated cell count, consisting of more than 85% degenerated neutrophils, and physiologically, a protein content of more than 30 g/L are reported [6]. Thoracocentesis enables the collection of diagnostic specimens and achieves patient stabilisation. The definitive diagnosis of feline pyothorax can be achieved by a cytological examination in combination with bacterial culture [5].

Currently, there is a lack of local data on the most common bacteria involved in feline pyothorax cases. This retrospective study aimed to determine the clinical manifestations, radiographic features, common concurrent conditions (where applicable), associated organisms and survival rate in feline pyothorax cases. This study will provide useful insights into feline pyothorax and help clinicians in the local setting in the selection of antimicrobials for feline pyothorax cases.

## 2. Materials and Methods

### 2.1. Patient Data

Medical records of cats diagnosed with pyothorax from 2013 to 2020 at University Veterinary Hospital, University Putra Malaysia were evaluated. Cases were included if their medical records were complete, two-view thoracic radiographs were consistent with feline pyothorax and bacterial growth was present on culture. Patient data of the cats with pyothorax including sex, breed, management (outdoor, indoor or semi-roamer), single or multi-cat household, vaccine history, number of days with clinical signs before presentation, underlying or concurrent disease and medical history were recorded and analysed. Body temperature, weight, body condition score (BCS) and presenting signs of the cats were also evaluated. In addition, complete blood count and serum biochemistry results were retrieved and evaluated where available.

### 2.2. Radiographic Findings

Patients’ radiographs were reviewed and evaluated systematically by two veterinarians to reach a consensus using a systematic checklist as suggested in previous study [20]. The checklist consists of abnormalities of extrathoracic structures, pleural space, pulmonary parenchyma and mediastinum (Table 1).

### 2.3. Pleural Fluid Analysis and Cytological Evaluation

Data of the cytological evaluation of the pleural effusions were retrieved. Turbidity was graded subjectively from 0 to 4+, with 0 as crystal clear and 4+ being the most turbid until newsprint could not be read through the tube. Specific gravity of the specimens was measured using a refractometer. The erythrocyte, protein and leukocyte levels of the pleural effusions were graded semi-quantitatively (negative, 1+ to 4+ scale) using commercial reagent dipsticks (LabStrip U11Plus, 77 Elektronika Kft., Budapest, Hungary) that undergo colour change reaction relative to concentration, which were later read by electronic analyser (DocUReader 2 Pro, 77 Elektronika Kft.). Pleural effusion smear samples stained with Wright’s stain and Gram stain were examined microscopically for cellular abnormality, presence of neoplastic cells and infectious agents such as fungal hyphae, yeast cells parasites and/or bacteria.

### 2.4. Bacterial Culture and Antimicrobial Susceptibility

Bacterial culture results and antimicrobial susceptibility profiles of pleural effusion specimens that were collected via thoracocentesis were retrieved. The type of infection (either bacterial infection of single or mixed species) was recorded. The samples were cultured on 5% horse blood and MacConkey agar plate incubated for 24 to 48 h at 37 °C in aerobic conditions. Bacteria were identified by performing conventional biochemical tests and the antimicrobial susceptibilities were determined via the Kirby Bauer technique [21].

### 2.5. Therapy and Outcomes

All cats in the study that received antimicrobial(s) treatment were assessed for recovery and outcome based on subsequent visits to the hospital. Owners of the cats were contacted via telephone to obtain information of post-treatment outcomes in cases where they did not return for follow-up appointments. The data were presented as descriptive statistics. The association between survival rate and chest tube placement was analysed using Fisher’s exact test. Analysis was achieved using a web-based Fisher’s exact test (https://www.socscistatistics.com/tests/fisher/default2.aspx) (accessed on 8 July 2021), *p*-value of less than 0.05 was considered statistically significant.

## 3. Results

### 3.1. Signalment and History

Twenty-eight cats were diagnosed with pyothorax between 2013 and 2020 and the age of the cats ranged from 4 months to 10 years (median 11 months, mean 23 months), with 16 females (all intact) and 12 males (8 intact males, 4 castrated males). Of these 28 cats, 21 were domestic short hair, 2 were Persian crosses, 4 were pure Persians and 1 was Siamese. The median 5-point body condition score of the cats was 2 with a mean body weight of 2.43 kg. Patient signalment, vaccination status, and the management of the patients are presented in Table 2.

From the history of the cats retrieved from owners, concurrent conditions were identified in 15 cats with pyothorax (53.6%), as seen in Table 3. Seven cats with feline pyothorax (25%) had concurrent feline upper respiratory disease. The cats had clinical signs of upper respiratory infection such as ocular or nasal discharge, sneezing, conjunctivitis or had a history of chronic flu. Direct inoculation of bacteria was suspected in four cats (14.3%). The cats either had healed thoracic wall puncture wounds, cat fight history or the presence of a gangrenous swollen wound resulting from a cat bite. Four cats (14.3%) had other concurrent conditions or diseases, where one cat was jaundiced, one had diabetes mellitus, one cat was blind and one cat was positive for feline immunodeficiency virus and feline leukaemia virus. One cat (3.6%) had a diaphragmatic hernia due to a motor vehicle accident.

### 3.2. Clinical Findings

The mean body temperature during presentation of the cats was 38.3 °C (*n* = 25), with a range of 35.6 °C to 41.2 °C. At clinical examination, respiratory signs were the predominant findings compared to other non-specific clinical findings in sick cats. The most frequent clinical presentations were dyspnoea (75.0%), harsh or dull lung sound (75.0%), open-mouth breathing (64.3%), abdominal breathing (57.1%), tachypnoea (39.3%), lethargy (39.3%), anorexia (39.3%), dehydration (35.7%) and ocular or nasal discharge (28.6%). Other non-specific clinical findings that were documented were pale mucous membranes (25.0%) and pyrexia (17.9%) (Table 4). The cats developed clinical signs such as respiratory distress and anorexia one to 30 days (mean of six days) before presentation.

### 3.3. Haematology and Biochemical Results

Only 27 cats had their haematological and biochemical results evaluated and their means and medians are presented in Table 5. Of the 27 available haematological results, the most significant abnormalities were monocytosis (68%), increased band neutrophils (68%), increased segmented neutrophils (64%), leucocytosis (61.5%), hyperglobulinaemia (65.4%) and hyperproteinaemia (57.7%).

### 3.4. Radiographic Findings

**Figure 1:**
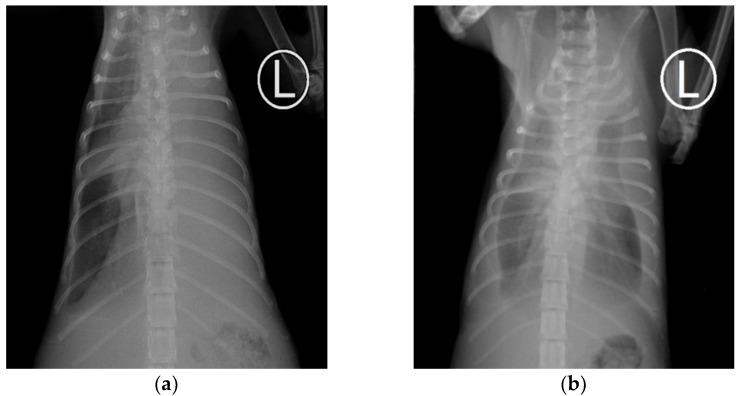
Ventrodorsal radiographs of feline pyothorax: (**a**) Unilateral pleural effusion; (**b**) Bilateral pleural effusion.

The radiographic findings of 28 cats with pyothorax are summarised in Table 6. As expected, pleural effusion (Figure 1) was the most typical finding in thoracic radiographs of feline pyothorax, present in all cases (100%), with bilateral pleural effusion (67.9%) more frequently found than unilateral pleural effusion (32.1%). Lung consolidation (75%), obscured cardiac silhouette (71.4%), hepatomegaly (32.1%), peritoneal effusion (10.7%), sternal lymph node enlargement (7.1%) and cranial mediastinal lymph node enlargement (3.6%) were other notable findings on radiographs.

### 3.5. Pleural Fluid Analysis and Cytological Evaluation

Pleural fluid analysis was performed in 19 out of 28 cats with pyothorax. For the appearance of the samples, four were light yellow, seven were milky or creamy light yellow, one was milky light reddish, two milky or whitish, four were pale reddish, one brown reddish and two brownish yellow. One cat had yellow gel-like pleural fluid and tested positive for coronavirus antibodies via an enzyme-linked immunosorbent assay. The pH of these samples ranged from 5.5 to 8 (mean: 7.0) and the specific gravity ranged from 1.025 to 1.051 (mean: 1.035). All samples fulfilled the criteria of exudate, which is defined by having a specific gravity of more than or equal to 1.025 and constituting predominantly degenerated neutrophils [22]. An erythrocyte level of 3+ to 4+ (median: 4+), protein level of 2+ to 4+ (median 4+), leucocyte level of 1+ to 3+ (median 3+), and turbidity level of 1+ to 4+ (median 4+) were observed in these samples.

**Figure 2:**
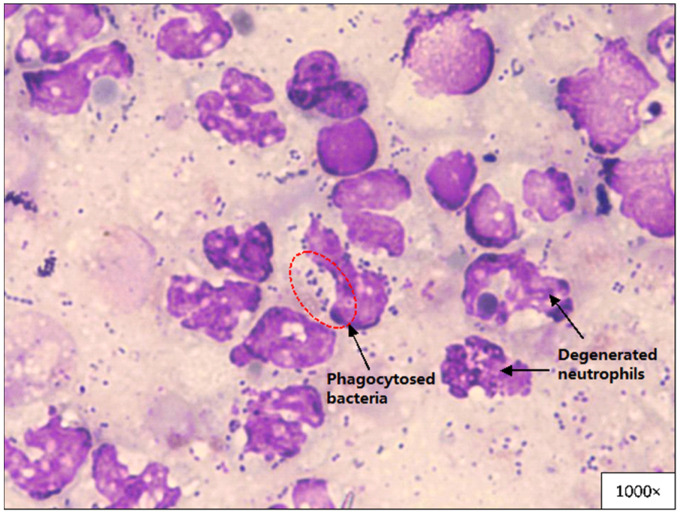
Phagocytosed bacteria and degenerated neutrophils in feline pyothorax pleural effusion sample (Wright’s stain).

Septic effusions that consist of phagocytosed and free bacteria (coccobacillus/cocci/mixed type) were the most common findings in nine (47.4%) out of 19 pleural effusion samples that were examined cytologically. Degenerated neutrophils were the most common cell types observed in 15 samples (78.9%) (Figure 2). Neoplastic cells, fungal hyphae, yeast cells and parasites were not observed in any of the samples.

### 3.6. Bacterial Identification and Antimicrobial Susceptibility

Aerobic bacteria isolated from 28 feline pyothorax cases are presented in Table 7. The bacteria isolated ranged from 1+ to 4+ (mean of 2+). Overall, the most common bacteria identified were *Pasteurella multocida*, *Streptococcus canis* and *Escherichia coli*. Only three cases (10.7%) had a mixed infection from multiple bacteria, while the rest of the 25 cases (89.3%) were due to a single bacterial species. In terms of bacterial infections of single species, *Pasteurella multocida* still remained the most common bacterium found. Antimicrobial susceptibility tests performed in 16 of the cases are presented in Table 8. Marbofloxacin demonstrated the highest antimicrobial resistance (71.4%) in 14 samples. Poor susceptibility was observed with cephalexin at 61.5% for 13 samples. Amoxicillin–clavulanate showed the lowest antimicrobial resistance at 23.5% (four out of 17 samples).

### 3.7. Therapy and Outcomes

Cats with acute and dyspnoeic presentation were placed in a concentrated oxygen chamber for initial stabilisation. Thoracocentesis or chest tube placement was performed to withdraw the pleural fluid from the cats. Parenteral antimicrobial therapy, intravenous fluid therapy, and serial thoracic radiographs for reassessment of the condition were part of the management of these cases. Antimicrobials were administered for a range of 8 to 30 days (mean 14 days). Among 28 cats with pyothorax, 18 cats (64.3%) had chest tube placement, while 10 cats (35.7%) had intermittent thoracocentesis. Chest tubes were placed for four to eight days (mean of six days), excluding cases that died before the removal of the chest tube. All cats were treated with a combination of antimicrobial drugs, using more than one route of administration, and a wide range of dosages were used, as presented in Table 9. Based on the available data on the use of antimicrobials in this study, 23 cats were treated with two antimicrobials and five cats with three antimicrobials during the pre-diagnosis management of the cases. One cat died acutely on the day of presentation. The outcomes according to the pre- and post-diagnosis antimicrobials used are presented in Table 10.

Successful treatment was defined as the absence of recurrence at the time of re-evaluation two weeks after the last pyothorax treatment. Of the 28 cats that had pyothorax, 13 cats survived (46.4%) and the other 15 cats died (53.6%) at time of re-evaluation. Among the 18 cats that had chest tube placement, 12 cats (66.7%) survived. Comparatively, the survival rate was lower in cats that did not have a chest tube placed, where only one (10%) out of 10 cats survived. The survival rate of the cats with chest tube(s) was statistically significant (*p* = 0.006). Both unilateral and bilateral types of pleural effusion had similar survival rates at 44.4% and 47.4%, respectively. The most successful combination of treatments with good prognosis was the usage of chest tube lavage with sterile 0.9% NaCl solution. The outcomes for 28 cats are presented in Table 11. From the 18 cats that had chest tube drainage, 11 cats (61.1%) had complications as presented. Among 11 cats that developed complications, two (18.2%) cats did not survive. The most common complications seen among the cats were the development of lung bullae (27.3%) and subcutaneous emphysema (27.3%) (Table 12). From the clinical records, there were eight cases that did not turn up for scheduled check-up.

## 4. Discussion

In this study, 28 cases of feline pyothorax were reviewed. The age of recruited cats in this study ranged from 4 months to 10 years (mean of 23 months), which was younger than that in previous studies [7,8,14]. Male and female cats were equally represented, while domestic short hair cats were over-represented in this study. It has also been reported in previous studies that there is no breed or sex predisposition for feline pyothorax [22].

Most of the pyothorax cats in this study were from multi-cat households (60.7%) and had access to the outdoors (64.3%). Both multi-cat households and the outdoor environment increase the chance of contact and exposure to other cats. This increases the risk of upper respiratory disease transmission from one cat to another. The aspiration of oropharyngeal flora, subsequent colonisation of the lower respiratory tract and direct extension of infection from bronchi and lungs was the most common mechanism proposed in feline pleural space infection [7], human empyema [23] and equine pleuropneumonia [24]. Out of the 28 pyothorax cats, seven (25%) were found to have concurrent feline upper respiratory infection. Similar findings were found in another study where 30% of the pyothorax cats also had typical history or clinical signs of upper respiratory tract infection [7]. Nearly 80% of the pyothorax cats in this study did not have updated vaccination status against feline herpesvirus and feline calicivirus, making them vulnerable to feline upper respiratory disease. Immunosuppression from the initial upper respiratory virus infection may allow secondary bacterial infection that may invade through compromised lung parenchyma, resulting in bacterial pneumonia. The inflammatory response will initiate the release of oedematous and exudative fluid, protein and neutrophils into the pleural space. Extensive complex parapneumonic diffusion may develop into pyothorax when the formation of pus is sufficient [5,9,22]. These findings suggest the antecedent role of upper respiratory infection in pyothorax.

Cats that live in multi-cat households or those that have outdoor access would experience greater inter-cat aggression, and thus a greater chance of fighting and biting, leading to puncture wounds. Thoracic puncture wounds were the second most common concurrent condition among the pyothorax cats. Cats with a history or presence of thoracic puncture wounds were only identified in four cases (14.3%), which is similar to findings of a large-scale retrospective study of 80 cats [8]. The result of this study contests the widespread belief that direct inoculation of the pleural cavity by bite wounds through the thorax is the most common aetiology of feline pyothorax. The direct inoculation of oral flora such as *Pasteurella* spp. into the thorax is possible via a bite wound. The bacteria are introduced into the thoracic cavity, causing septic inflammation. Viruses such as feline immunodeficiency virus (FIV) and feline leukaemia virus (FeLV) can also be transmitted through biting and may cause immunosuppression. Taking into consideration both facts, the association between retroviruses and pyothorax and the immunosuppressive effect of FIV and FeLV might be worth investigating. Previous studies in the United States, the UK, America and Australia reported that 2.86–5.88% pyothorax cats were FIV positive, while 4.35–4.41% pyothorax cats were FeLV positive [8,22]. Given the fact that not all the cats in this study underwent viral testing and only one cat was positive for FIV and FeLV, an association between FIV, FeLV and feline pyothorax was not able to be drawn.

Four cats (13.8%) had other concurrent conditions that may also compromise the immune response of the cats. One cat tested positive for FIV and FeLV. Two cats had systemic conditions or diseases such as jaundice and diabetes mellitus, respectively, while another cat was blind and malnourished since it was young. These conditions may suppress their immune system, making them vulnerable to secondary bacterial infection and increasing their chance of developing pyothorax.

The duration of clinical signs of pyothorax cats prior to diagnosis is very much dependent on the owner’s observation. Clinical signs were observed one to 30 days before presentation (mean of six days), similar to a previous report by Barrs et al. [7]. Dyspnoea (75%), harsh or dull lung sounds (75%) and open-mouth breathing (64.3%) were the most common clinical signs among the pyothorax cats. Non-specific clinical signs of feline pyothorax such as inappetence, lethargy, poor body condition, dehydration and, less frequently, coughing are often subtle and go mostly unnoticed by owners. Dyspnoea, which was commonly observed in the present study, is in contrast to another study that reported dyspnoea was overlooked by up to 40% of the owners of pyothorax cats [9]. Pleural effusion has to become severe enough to give rise to respiratory distress for owners to notice a problem [8]. Furthermore, cats tend to hide clinical signs of the disease well, showing minimal signs of pain and discomfort in the presence of humans, other animals or in stressful conditions, making it difficult for owners to notice these subtle signs of disease. Most of the cats in this study had access to the outdoors or lived in a multi-cat household. Thus, picking up subtle changes or abnormalities in these cats would prove to be harder for owners. Besides, it is also possible that the owners were not aware of the clinical signs of pyothorax and what to look out for.

Complete blood count and serum biochemistry analysis are part of the minimum database to access the general condition of the patient and guide therapeutic management of feline pyothorax. Monocytosis (68%) and leucocytosis (61.5%) were among the most common abnormal haematological findings in this study, similar to what has been reported previously. Monocytes are released into the circulatory pool as an immune response towards chronic inflammation or stress [25,26]. Only one cat had degenerative left shift where the band neutrophil count exceeded the segmented neutrophils. The cat had a poor prognosis and was dead within 24 h after presentation. Serum biochemical parameters such as hypoalbuminemia (40.7%) and hyperglobulinaemia (65.4%) were also observed. Increased permeability of vessels due to inflammation causes albumin to escape into the pleural cavity, resulting in hypoalbuminemia [27]. During acute phase reaction, hepatic production of albumin is decreased and that of α- and β-globulin is increased, causing hyperglobulinaemia. Only nine out of 27 cases showed an albumin to globulin ratio (A:G) that was lower than 0.5, though more cases with such findings were expected due to inflammation in feline pyothorax. The explanation of this finding would be that the majority of the cats were dehydrated and therefore had a greater albumin level, influencing the A:G ratio. In 10 cases, urea was increased by less than one-fold, which may be explained by pre-renal azotaemia. This condition can be explained by the dehydration condition faced by the pyothorax cats, similarly to the previous report of pyothorax cats [28]. There was also one case that had a one-fold increment and another case with a two-fold increment, respectively. Similarly, mild non-regenerative anaemia, mild lymphocytosis, severe neutropenia, thrombocytopenia and slightly elevated urea have also been reported in another study [29]. The complete blood count and serum biochemistry findings of these cases are general findings of inflammation and not specific for pyothorax.

Thoracic radiography is indicated in cases of animals that present with breathing difficulty. In total, 67.9% of the cats in this study had bilateral effusion, similar to various previous studies [7,8,14]. Pleural fluid may remain unilateral if the pleural fluid is too viscous, the mediastinal pleura is not fenestrated or fenestration becomes closed due to inflammation or plugging [15,30,31]. Thus, unilateral effusion may remain unilateral or progress to a bilateral distribution. The main significance of unilateral or bilateral effusion is in the treatment. A unilateral chest tube is indicated when unilateral effusion is present and if effective drainage can be accomplished, leaving minimal residual effusion with only one tube [13]. Bilateral chest tubes are indicated in cases of bilateral effusion [10]. Bilateral chest tubes are more likely to give effective drainage in cases of persistent loculation of fluid [32].

In comparison to radiography, computed tomography would yield a more detailed assessment of underlying parenchymal and pleural abnormalities, avoid the superimposition of images, provide details with regard to severity and provide an opportunity for the assessment of mediastinal, sternal and tracheobronchial lymph node enlargement [9]. A previous study reported that computed tomographic attenuation values can be a possible predictor of the type of pleural fluid [33,34]. Nevertheless, this method might not be feasible and can be inconsistent in sensitivity due to the technological and technical difference of the respective computed tomography scanner used. The use of computed tomography as a diagnostic tool would be also risky as the patient has to undergo general anaesthesia and often these patients suffer from respiratory compromise. On the other hand, thoracic ultrasonography is a diagnostic tool that is able to detect moderate to large volumes of pleural effusion without imposing anaesthetic risk on patients with respiratory compromise [13]. Pulmonary foreign bodies, intrathoracic masses, and pulmonary abscesses can sometimes be detected via ultrasonography. Ultrasound-guided thoracocentesis is a tool for both diagnosis and treatment, allowing the evaluation of pleural fluid and the drainage of the pleural fluid. Although grass awns were not recorded in any of the cases in the present study, migrating grass awns can be a potential cause of pleural effusion in cats. However, radiographic changes are not very sensitive for such migrating foreign bodies. Computed tomography and bronchoscopy are more sensitive to diagnose them [35].

Pleural fluid analysis and cytology evaluation in this study reported a mean specific gravity of 1.025 to 1.051 (mean 1.035), septic effusions that consisted of phagocytosed and free bacteria (coccobacillus/cocci/mixed type) were found in nine (47.4%) cases and degenerated neutrophils were observed in 15 samples (78.9%). The results in this study are similar to the pleural fluid analysis in previously reported cases in cats [7,14]. Gram staining and cytological examination of pleural fluid allows quick assessment for the presence of bacteria and this assessment can assist the selection of empirical antimicrobial treatment before a culture and susceptibility result is out [22].

Cytologically, bacteria were seen in nine out of 17 samples (47.4%), while all samples (100%) had positive bacterial culture results. As shown in this study, the cytological visualisation of bacteria from pleural fluid specimens of feline pyothorax and results of bacterial culture may not always be consistent. Thus, bacterial culture should be performed in every case and cytological results should always be compared to the culture results [9].

The most common single bacterium involved in this study was *Pasteurella* spp., while mixed bacterial infections involved both *Staphylococcus* spp. (two cases) and *Streptococcus* spp. (two cases). Despite the majority of the cases involving single bacteria (89.3%), more bacteria were suspected to be involved in the cases. Taking the fact that these cases were presented at the hospital as referral cases, it is highly possible the cases had antimicrobial treatment before being referred. False negatives were also suspected if there was insufficient growth of certain isolates in vitro or fastidious bacteria such as anaerobes and *Mycoplasma* spp. [9]. A study reported that mixed isolates of oropharyngeal flora are often involved in feline pyothorax, primarily consisting of *Bacteroides* spp., *Clostridium* spp., *Streptococcus* spp., *Mycoplasma* spp., and *Pasteurella* spp. [10]. About 20% of feline pyothorax cases were caused by an infectious agent other than oropharyngeal flora, such as *Rhodococcus equi, Nocardia* spp., *Klebsiella* spp., *Proteus* spp. And *Pseudomonas* spp. [9]. Comparing pyothorax cases in cats and dogs, mixed microbial infections are common in both species [5,36]. Isolated Gram-negative, facultative anaerobic rods are predominantly non-enteric in origin in cats, while they have an enteric origin in dogs [9]. Similar to animal studies, mixed aerobic and anaerobic bacteria are commonly isolated from human patients. Aerobic organisms such as *Escherichia coli, Haemophilus influenza* and *Klebsiella pneumoniae* and the anaerobic bacterium *Bacteroides fragilis* are commonly isolated [37].

In general, the most common bacteria identified in this study were *Pasteurella multocida* (28.1%), *Streptococcus canis* (15.6%) and *Escherichia coli* (15.6%). *Pasteurella* spp. is a commensal bacterium and part of the natural flora of the feline nasopharynx and upper respiratory tract [38]. The inhalation of droplets secreted from the upper respiratory tract or infection via a bite wound are both possible routes of infection [39]. A study reported that *Streptococcus canis* has been isolated from the nasal cavity in up to 10% of cats suffering from chronic upper respiratory disease [40]. *Streptococcus canis* infection is usually opportunistic in older cats due to wounds, immunosuppression or viral infection and it is more prevalent with exposure to grass awns [41]. *Streptococcus canis* was the second most common isolate of the pyothorax cats. The findings in this study are in contrast to other studies that claimed that *Streptococcus canis* were not isolated in pleural fluid samples of pyothorax cats [14,36]. This could be explained by the stress faced by cats that are from a multi-cat household setting and are exposed to the outdoors, thus causing immunosuppression and allowing this opportunistic pathogen to thrive. *Escherichia coli* is a member of the *Enterobacteriaceae* family, which are known to be natural inhabitants of the gastrointestinal tract, oesophagus or mouth of dogs and cats [38]. *E. coli* is the most important bacterial pathogen of extraintestinal infection, including the urinary, respiratory and reproductive system in both dogs and cats [42]. In this study, *E. coli* were isolated in five out of 16 pleural samples (31.25%), contrary to studies that claimed that *Enterobacteriaceae* such as *E. coli* were relatively uncommon and rarely isolated (0–7%) [7,14,36]. *E. coli* is a well-recognised multi-sectoral indicator of antimicrobial resistance, and extraintestinal *E. coli* infections are often reported to be pathogenic in nature [43,44,45]. Thus, the detection of *E. coli* in this study raises concerns of antimicrobial resistance.

*Pasteurella multocida, Streptococcus canis* and *E. coli* are zoonotic bacteria. It has been reported that *P. multocida* infection may result in septicaemia, meningitis, brain abscess, pneumonia, endocarditis, and systemic infection [38]. A woman was reported to experience septic shock, sinusitis and pneumonia from *P. multocida* post cat exposure and cat scratches [46]. A *P. multocida* isolate was recovered from the urine of a woman with a urinary tract infection, showing molecular evidence of zoonotic transmission from the patient’s cat [47]. The zoonotic transmission of *P. multocida* is possible via aerosol inhalation and contact with pet secretion. Another study reported a case of a geriatric woman with pneumonia that resulted from non-traumatic transmission of *P. multocida* from a domestic dog [48]. *S. canis* is a member of Lancefield group G streptococcus and it could be pathogenic to humans, since their physiological and biological characteristics are very much like those of group A streptococci. It is also interesting to note that 47% of group G streptococci isolates from cats were reported to be capable of growing in human blood [49]. *E. coli* consist of various strains that are distinctively different epidemiologically and phylogenetically, affecting any organ or anatomical site. In humans, *E. coli* are capable of causing meningitis, septicaemia and urinary tract infections. Animals are recognised as crucial reservoirs of potential sources of extraintestinal pathogenic *E. coli* [44,50].

In the human context, *Streptococcus* spp. (e.g., *S. intermedius*), *Staphylococcus* spp. (e.g., *S. aureus*), and Gram-negative aerobes such as *E. coli* and *Haemophilus influenzae* were cultured from human community-acquired and hospital-acquired pleural infection. Similarly, some isolates were also found in cats [7,51]. Human empyema, which was caused by staphylococci, enterobacteria, enterococcus and *Pseudomonas aeruginosa*, has been reported in the United Kingdom [52].

This study has highlighted a major concern of antimicrobial resistance in feline pyothorax cases. Only five (amoxicillin–clavulanate, ceftriaxone, sulfamethoxazole/trimethoprim, norfloxacin, tetracycline) out of 16 antimicrobials tested had a susceptibility of above 50%, but the sample size was limited. The exposure of bacteria to antimicrobials may lead to the selection of resistance; therefore, identification and antimicrobial susceptibility testing are recommended in every case of feline pyothorax. Among the antimicrobials that underwent susceptibility testing, amoxicillin–clavulanate was susceptible in 13 out of 17 samples (76.5%). This study provided evidence of amoxicillin–clavulanate being the antimicrobial of choice against aerobic bacteria that were isolated in this study while pending the result of culture and antimicrobial susceptibility testing.

Comparing the antimicrobial susceptibility across the three most common bacteria isolated in the cases, namely *P. multocida*, *E. coli*, and *S. canis*, amoxicillin–clavulanate remained the recommended antimicrobial of choice against aerobes in this study. On average, amoxicillin–clavulanate demonstrated an antimicrobial susceptibility of 50% to 100% towards the bacteria mentioned earlier, but the sample size was limited. Enrofloxacin is the antimicrobial often chosen in cases of feline pyothorax at veterinary clinics in Malaysia. Although enrofloxacin has been recommended to be added as an antimicrobial for improved Gram-negative coverage while pending culture results, antimicrobial susceptibility testing in this study revealed that eight out of 14 samples tested against enrofloxacin were resistant. Thus, this again is of major concern and enrofloxacin antimicrobial resistance should be taken into consideration by clinicians.

Different levels of antimicrobial susceptibility have been reported in various countries. In a comprehensive study across Europe, antimicrobial susceptibility levels of 54.6% to 100% were reported across different antimicrobials and bacteria [53]. Another retrospective study of pyothorax in the United States revealed an antimicrobial susceptibility of 80 to 100% towards *Pasteurella multocida* and *Escherichia coli* [36]. The level of antimicrobial resistance was different than what was found in our study. Although antimicrobial resistance is largely attributed to indiscriminate use as growth promoters in farm animals [54,55] and the widespread use of quaternary ammonium disinfectants [56], nevertheless, the unreasonable use of antibiotics by pet owners, the limited surveillance and control of antimicrobial usage in companion animals, and the paucity of data on local antimicrobial resistance profiles and guidelines on rational antimicrobial usage in companion animals may have also contributed to the emergence of antimicrobial resistance [57,58]. The use of different methods for antimicrobial susceptibility testing and different breakpoints for the evaluation of the results in different countries may vary greatly, thus resulting in different levels of antimicrobial susceptibility according to the country [57].

In this study, among the combination of antimicrobials used, metronidazole was the most common antimicrobial used (21 out of 28 cases), followed by amoxicillin–clavulanate (14 out of 28 cases) and marbofloxacin (13 out of 28 cases). Our choice of antimicrobials for pyothorax is in line with Australasian Infectious Disease Advisory Panel Antibiotic Prescribing Detailed Guidelines and Antimicrobial Guidelines Working Group of the International Society for Companion Animal Infectious Disease [16,59]. Some studies stated that empiric therapy for pyothorax with Gram-negative facultative bacteria should include aminoglycosides (gentamicin or amikacin) or fluoroquinolones [36,60]. The usage of aminoglycoside was discouraged by another study due to poor penetration into the pleural space and potential nephrotoxic and ototoxic effects [61]. Thus, aminoglycoside was not the choice of antimicrobial utilised by the clinicians in this study.

Antimicrobial selection should be based on the identification and susceptibility of the organism in every case of feline pyothorax to prevent the development of antimicrobial resistance, which was seen in these cases. Isolation, the identification of bacteria via biochemical tests and antimicrobial susceptibility testing take a minimum of 3 days. Based on the antimicrobial susceptibility testing, amoxicillin–clavulanate showed the lowest antimicrobial resistance at 23.5% (four out of 17 samples). Amoxicillin–clavulanate is the recommended antimicrobial against the aerobic bacteria that were found in this study and it is to be given in combination with antimicrobials that cover anaerobic bacteria. Awareness and knowledge of antimicrobial usage and antimicrobial resistance should be promoted. In Malaysia, pet owners tend to visit other clinics easily and demand different treatment for their pet when the owners do not see an improvement in a short period of time. Veterinarians might feel stressed as owners would question the veterinarians’ competency in treating the patients when the owners’ expectations were not met. Antimicrobial resistance may occur when the owner does not comply with the treatment plan, fails to administer the antimicrobial at the correct dosage or frequency, fails to complete the initial antimicrobial course, or when a new type of antimicrobial is introduced by the second vet.

Intravenous antimicrobials were administered as initial and ongoing treatment and then substituted with oral antimicrobials after the patient was eating well or discharged from hospital in this study, similar to what has been proposed in a previous study [32]. The patients were treated with antimicrobials with a range of 8 to 30 days (mean duration of 14 days). Antimicrobials in this study were dispensed weekly together with a scheduled re-evaluation after the last treatment. Unfortunately, eight owners in this study did not turn up for the scheduled check-up, which may have resulted in antimicrobial treatments being stopped abruptly. Generally, a high dose of antimicrobials for extended periods of time such as oral antimicrobials for a minimum of 3 weeks and ideally a duration of 4–6 weeks is recommended as feline pyothorax is associated with devitalised tissue and relapse is common if treatment is discontinued too early [7,36,41,60].

All 28 cats in this study were treated with a combination of antimicrobials. Three cats from combination number 4 (amoxicillin–clavulanate + marbofloxacin + metronidazole) and four out of six cats from combination number 10 (marbofloxacin + metronidazole) recovered from pyothorax. All recovered cats in both combinations had at least one antimicrobial that had coverage against aerobic bacteria and another for anaerobic bacteria. All recovered cats that were treated with those antimicrobial combinations also had chest tube placements. From these findings, it is hypothesised that the usage of a combination of antimicrobials with coverage for aerobes and anaerobes together with chest tube placement could have a significant impact on the survival rates of pyothorax cats. Changing the antimicrobials based on the susceptibility testing result is also essential in achieving a better treatment outcome.

Chest tube placement is crucial in the treatment of pyothorax cases. This is evident in this study as all survivors (13 cats) had intermittent chest tube drainage as part of treatment (except one cat that survived without the introduction of a chest tube). In total, 18 out of 28 cats (64.3%) had chest tube placement and 12 cats (66.7%) that had a chest tube survived. Chest tubes were placed for an average of nine days, slightly more than the mean duration of five to seven days in other studies [7,8,14]. In general, the decision for chest tube removal was based on fluid production (reaching 3 to 5 ml/kg) and clinical, radiographic or pathological improvement, which may differ on a case-by-case basis. Chest tube drainage, antimicrobial treatment and supportive care as medical therapy performed in cats in this study are considered standard treatments for cases of feline pyothorax [5,32]. Furthermore, the survival rate of cats with chest tube placement was significantly (*p* = 0.006) improved compared to those without. This might suggest that the improved survival rate of pyothorax cats is associated with chest tube placement, similar to what has been proposed in other studies [13].

Feline pyothorax is a serious life-threatening condition where in this study only 13 out of 28 cats (46.4%) survived, similar to what has been reported earlier [8]. Both bilateral and unilateral involvement of pleural effusion in this study showed similar survival rates (around 50%). The number of cases observed in this study is too small to draw conclusions on the relationship between the laterality of pleural effusion and survival rate. Bilateral effusion is distinctly associated with increased mortality, especially in cases of community-acquired pneumonia in humans [5,62]. It will be worth investigating the possibility of the laterality of pleural effusion as a prognostic factor in feline pyothorax.

Thoracic lavage with warm isotonic saline was performed in 14 out of the 18 cats with chest tubes. The risk and benefits of chest tube lavage have been a topic of discussion in several veterinary publications. An improvement was seen in pyothorax cases with chest tube lavage, where a shorter chest tube placement was needed with thoracic lavage compared to those without [32]. On the contrary, another study claimed that the evidence of thoracic lavage influencing prognosis in animals with pyothorax is not well established [41]. Another recent study discouraged thoracic lavage in felines due to the risk [8]. Possible nosocomial infection from an unintentional breach of aseptic technique and the introduction of a large amount of fluid into the pleural space, followed by the inability to retrieve it, were risks from the thoracic lavage procedure [63].

Chest tube drainage is essential for successful recovery; nonetheless, there are some associated risks. Up to 61.1% of cases that had chest tube placement had complications similar to what has been reported previously [7,10]. The most common complications seen among the cats in this study were lung bullae (27.3%) and subcutaneous emphysema (27.3%). Despite 11 pyothorax cats having developed complications, only two pyothorax cats (18.2%) died. The underlying cause of lung bullae in this study was not identified. Lung bullae in cats are rare and have been described in association with bronchopulmonary dysplasia in a study [64]. Subcutaneous emphysema, which was seen in this study, might have been caused by the introduction and removal of the chest tube or leakage from the chest tube, as proposed in human literatures [65,66]. Pneumothorax cases (18.2%) that were noted in this study could have occurred due to ruptured or iatrogenic lung bullae after thoracocentesis as seen in another study [67]. Failure in recovering 75% or more instilled thoracic lavage solution was seen in 18.2% of the cases, which could be due to chest tube kinking, chest tube obstruction or the loculation of fluid pockets. To reduce the chance of kinking or obstruction, a thoracotomy tube of the greatest diameter that can fit comfortably between intercostal spaces should be selected [32]. Minimising the risk of pneumothorax may be attained by subcutaneous insertion of the chest tube through the skin two or more intercostal spaces caudal to the site where the tube enters the thoracic cavity [9]. Caution and constant monitoring should be practiced to prevent chest tube complications such as iatrogenic trauma when the chest tube is inadvertently removed or an infection from aseptic thoracocentesis.

Surgical thoracotomy is indicated in cases where medical therapy has failed, or when there are extensively loculated effusions on thoracic ultrasonography or post-drainage radiographs or when there is evidence of mediastinal or pulmonary abscesses on thoracic radiographs [14,68]. In this study, one cat underwent surgical thoracotomy with lung lobectomy (to remove a lung abscess). The surgical procedures were indicated as the cat’s condition was deteriorating despite the fact that medical treatment for pyothorax and surgical repair of concurrent diaphragmatic hernia were performed. Intercostal thoracostomy or median sternotomy for lavage, debridement and removal of the primary cause was not performed in other cases for the patients as it was not indicated, or the patient was too unstable to undergo general anaesthesia or due to the owner’s financial constraints.

The current study has several limitations. The risk factor was not analysed in this study as the number of cases in this study was limited and crucial information regarding the patient was limited. The diagnostic workup performed in this study was limited by clients’ financial constraints and the availability of laboratory equipment and was only performed upon clinicians’ request. Testing for viruses such as feline immunodeficiency virus and feline leukaemia virus was not performed for every patient; therefore, the association between pyothorax and virus status was undetermined in this study. The immunosuppressive potential of both the viruses in cats with pyothorax remains unknown. Having a diagnosis that includes the comprehensive history of the patient, viral testing and the inclusion of other diagnostic techniques such as polymerase chain reaction (PCR) will be useful and allows a more comprehensive investigation for prospective studies. The current study was unable to include the investigation of other plausible causes of pyothorax and emerging pathogens of respiratory interest in cat species, including fungal infection [7], respiratory mycoplasma [11], *Haemophilus* sp. [69], lungworm [70], larva migrans [7], anaerobes [14] and foreign body migration [13]. These pathogens can cause pyothorax directly or indirectly via parapneumonic extended infection into the pleural cavity. The role of these pathogens should be investigated in future prospective studies to provide an overall picture on the causative agents of feline pyothorax. It is worth mentioning that *Bordetella bronchiseptica* would have grown using the culture protocol in the present study, but it was not reported in any of the cases.

## 5. Conclusions

Identification and antimicrobial susceptibility testing of isolates from pleural samples provides useful information that is needed for the selection of antimicrobials. Amoxicillin–clavulanate has good antimicrobial susceptibility. Amoxicillin–clavulanate was the recommended antimicrobial against the aerobic bacteria that were found in this study and should be given in combination with antimicrobials that cover anaerobic bacteria. Epidemiology, virulence, pathogenesis and prognostic factors of pyothorax in cats will be worth investigating. Greater survival outcomes of cats were associated with initial stabilisation with oxygen supplementation and the combination of chest tube placement and aspiration, intravenous fluid and antimicrobial therapy. The definitive aetiology of feline pyothorax remains unidentified in most cases and ample room for future research exists in characterising the clinical presentation, specific radiological findings and predictive value of pleural fluid characteristics of feline pyothorax to establish a better understanding of the aetiopathogenesis of feline pyothorax in the Malaysian setting.

## Figures and Tables

**Table 1:** Radiographic thoracic abnormalities criteria.

Compartments	Checklist of Abnormalities
Extrathoracic structures	Discontinuity of diaphragm
	Loss of diaphragm outline
	Bite wound at cervical area
	Evidence of tumour/trauma
	Presence of subcutaneous emphysema
	Abnormal size of liver
Pleural space	Pleural effusion (bilateral or unilateral)
	Pleural thickening
	Presence of fluid or air or mass
	Pneumothorax
	Extra-pleural sign
Pulmonary parenchyma	Presence of atelectasis
	Presence of consolidation
	Presence of cavitary/mass opacity
	Presence of bullae
	Presence of pneumothorax
Mediastinum	
Cranial mediastinum	Presence of mediastinal widening
	Displacement/changes in size and contour of trachea
	Oesophageal abnormalities
	Pneumomediastinum
Middle mediastinum	Enlargement of tracheobronchial lymph nodes
	Cardiac size and shape (inclusive of vertebral heart size)
Caudal mediastinum	Displacement of aorta and oesophagus
	Border effacement of accessory lung lobe

**Table 2:** Patient signalment of 28 cats with pyothorax.

Patient Signalment	Number of Cases (*n*)	Percentage (%)
Male		
Intact Male	8	28.6
Castrated Male	4	14.3
Female		
Intact Female	16	57.1
Spayed Female	0	0
Vaccination		
Yes	6	21.4
No	22	78.6
Access to outdoors		
Yes	18	64.3
No	6	21.4
Not sure	4	14.3
Number of cats in household		
Single cat household	6	21.4
Multi-cat household	17	60.7
Not sure	5	17.9

**Table 3:** Concurrent conditions of pyothorax in 28 cats.

Concurrent Conditions	Number of Cases (*n*)	Percentage (%)
Concurrent feline upper respiratory disease *	7	25.0
Thoracic puncture wounds	4	14.3
Diaphragmatic hernia	1	3.6
Other concurrent condition/disease *	4	14.3
Unknown	13	46.4

* One cat had both concurrent feline upper respiratory disease and another condition (positive for feline immunodeficiency virus and feline leukaemia virus), classified into both categories.

**Table 4:** Clinical signs of 28 cats with pyothorax.

Clinical Signs	Number of Cases (*n*)	Percentage (%)
Dyspnoea	21	75.0
Harsh/dull lung sound	21	75.0
Open-mouth breathing	18	64.3
Abdominal breathing	16	57.1
Tachypnoea	11	39.3
Lethargy	11	39.3
Hyporexia/anorexia	10	35.7
Dehydration	10	35.7
Ocular/nasal discharge	8	28.6
Pale mucous membrane	7	25.0
Crackle lung sound	6	21.4
Pyrexia	5	17.9
Tachycardia	5	17.9
Cyanotic	5	17.9
Weight loss	4	14.3
Recumbent	3	10.7
Lymph node enlargement	3	10.7
Cough	2	7.1

**Table 5:** Haematological and serum biochemistry parameters of 27 pyothorax cats.

Parameters (Number of Samples)	Normal Reference Range	Mean ± SE	Median	*n* (%) with Value > Upper RL	*n* (%) with Value < Lower RL
Erythrocytes (RBC) (×10^12^/L) (*n* = 26)	5–10	7.01 ± 0.33	7.07	0 (0)	2 (7.7)
Haemoglobin (g/L) (*n* = 27)	80–150 g/L	102.05 ± 4.41	101.85	0 (0)	4 (14.8)
PCV (L/L) (*n* = 26)	0.24–0.45	0.28 ± 0.01	0.29	0 (0)	5 (19.2)
CWCC (×10^9^/L) (*n* = 26)	5.5–19.5	26.79 ± 3.43	24.05	16 (61.5)	3 (11.5)
Band neutrophils (×10^9^/L) (*n* = 25)	<0.3	1.40 ± 0.37	0.79	17 (68)	0 (0)
Segmented neutrophils (×10^9^/L) (*n* = 25)	2.5–12.5	20.31 ± 2.74	22.14	16 (64)	3 (12)
Lymphocytes (×10^9^/L) (*n* = 25)	1.5–7.0	3.46 ± 0.52	2.23	3 (12)	7 (28)
Monocytes (×10^9^/L) (*n* = 25)	0.2–0.8	1.62 ± 0.23	1.5	17 (68)	2 (8)
Eosinophils (×10^9^/L) (*n* = 25)	0.1–1.5	0.66 ± 0.19	0.22	3 (12)	7 (28)
Basophils (×10^9^/L) (*n* = 7)	Rare	0.0025 ± 0.0025	0	-	-
Platelets (×10^9^/L) (*n* = 24)	300–700	228.20 ± 34.92	206	0 (0)	17 (70.8)
Nucleated erythrocytes/100WBC (*n* = 8)	Rare	5.5 ± 1.83	3.5	-	-
Reticulocytes/100RBC (*n* = 16)	0.5–1.5	2.91 ± 1.16	0.5	6 (37.5)	7 (43.8)
Plasma Protein (g/L) (*n* = 26)	60–80	86.85 ± 2.67	85	15 (57.7)	0 (0)
Icterus Index (Unit) (*n* = 22)	<10	4.30 ± 1.18	2	2 (9.1)	0 (0)
Sodium (mmol/L) (*n* = 21)	146–156	149.40 ± 1.66	148.4	3 (14.3)	6 (28.6)
Potassium (mmol/L) (*n* = 20)	3.9–5.5	4.89 ± 0.17	4.95	4 (20)	2 (10)
Chloride (mmol/L) (*n* = 21)	110–132	112.62 ± 1.49	112.3	0 (0)	5 (23.8)
Calcium (mmol/L) (*n* = 2)	2.2–2.9	2.15 ± 0.05	2.15	0 (0)	1 (50)
Inorganic phosphate (mmol/L) (*n* = 5)	1.1–2.8	2.07 ± 0.23	2.4	0 (0)	0 (0)
Urea (mmol/L) (*n* = 27)	3.0–10.0	10.62 ± 1.11	8.8	13 (48.1)	0 (0)
Creatinine (mmol/L) (*n* = 27)	60–193	79.04 ± 5.48	80	0 (0)	8 (29.6)
ALT (U/L) (*n* = 27)	10–90	64.62 ± 16.89	41.6	2 (7.4)	0 (0)
GGT (U/L) (*n* = 3)	<6.0	3.33 ± 2.03	3	1 (33.3)	-
Total Protein (g/L) (*n* = 27)	55–75	78.74 ± 3.06	76	15 (55.6)	1 (3.7)
Albumin (g/L) (*n* = 27)	25–40	25.64 ± 0.82	25.4	0 (0)	11 (40.7)
Globulin (g/L) (*n* = 26)	25–45	53.14 ± 3.22	48.6	17 (65.4)	0 (0)
A:G (*n* = 27)	0.5–1.4	0.52 ± 0.03	0.5	0 (0)	9 (33.3)

*n* = number of samples; RL = reference limit; RL = red blood cell; PCV = packed cell volume; CWCC = complete white cell count; WBC = white blood cell; ALT = alanine transaminase; ALP = alkaline phosphatase; GGT = gamma glutamyl transferase; AST = aspartate transaminase; TP = total protein; A:G = albumin:globulin ratio.

**Table 6:** Summary of radiographic findings of 28 cats with pyothorax.

Radiographic Findings	Number of Cases (*n*)	Percentage (%)
Pleural effusion	28	100
Unilateral	9	32.1
Bilateral	19	67.9
Lung consolidation	21	75.0
Right		
-Cranial	14	50
-Medial	12	42.9
-Caudal	7	25
Left		
-Cranial	12	42.9
-Caudal	5	17.9
Obscured cardiac silhouette	20	71.4
Hepatomegaly	9	32.1
Peritoneal effusion	3	10.7
Vascular pattern	2	7.1
Cranial mediastinal lymph node enlargement	1	3.6
Sternal lymph node enlargement	2	7.1
Tracheobronchial lymph node enlargement	0	0

**Table 7:** Bacteria cultured from 28 cases of cats with pyothorax.

Bacteria Cultured	Number of Cases (*n*)	Percentage (%)
*Pasteurella multocida*	9	28.12
*Streptococcus canis*	5	15.63
*Escherichia coli*	5	15.63
*Streptococcus viridans*	2	6.25
*Staphylococcus pseudintermedius*	2	6.25
*Streptococcus equi*	1	3.13
*Staphylococcus intermedius*	1	3.13
*Methylobacterium mesophilicum*	1	3.13
*Aggregatibacter actinomycetemcomitans*	1	3.13
*Neisseria zoodegmatis*	1	3.13
*Enterobacter cloacae*	1	3.13
*Acinetobacter lwoffii*	1	3.13
*Pseudmonas aeruginosa*	1	3.13
Fungus-like bacteria	1	3.13
Total number of bacteria identified	32	

**Table 8:** Summary of antimicrobial susceptibility tests for 16 cases of cats with pyothorax.

Antimicrobials (Number of Samples)	Disk Content (µg)	Resistance (R + I)	Susceptibility (S)
Amoxicillin (*n* = 4)	10	3	1
Amoxicillin–clavulanate (*n* = 17)	30	4	13
Ampicillin (*n* = 1)	10	1	0
Azithromycin (*n* = 2)	15	1	1
Cephalexin (*n* = 13)	30	8	5
Ceftriaxone (*n* = 8)	30	3	5
Clindamycin (*n* = 1)	2	1	0
Enrofloxacin (*n* = 14)	5	8	6
Erythromycin (*n* = 2)	15	1	1
Gentamycin (*n* = 5)	10	3	2
Marbofloxacin (*n* = 14)	5	10	4
Norfloxacin (*n* = 1)	10	0	1
Sulfamethoxazole/trimethoprim (*n* = 3)	25	1	2
Tetracycline (*n* = 9)	30	4	5

**Table 9:** Summary of the details of antimicrobial administration in 28 cats with pyothorax.

Antimicrobial	Number of Cases	Route Administration	Dose (mg/kg)
Metronidazole	21	IV/PO	10
Amoxicillin–clavulanate	14	SQ/PO	12.5
Marbofloxacin	13	IV/PO	2
Cephalexin	4	PO	20
Ceftriaxone	3	IV	25
Enrofloxacin	2	IV	5

IV = intravenous; PO = per oral; SQ = subcutaneous.

**Table 10:** Summary of antimicrobials used in the treatment of 28 pyothorax cats.

Number	Pre-Diagnosis (Antimicrobials)	Post-diagnosis (Antimicrobials)	Number of Cats (*n* = 28)	Outcome (*n* = 28)	Remarks
1	Amoxicillin–clavulanate + metronidazole	-	10	6 died, 2 euthanised, 2 recovered	Six cats died in pre-diagnosed treatment. Two cats were euthanised due poor response to treatment. Two cats recovered in pre-diagnosed treatment.
2	Amoxicillin–clavulanate + metronidazole	Remained amoxicillin–clavulanate + metronidazole	1	1 recovered	Bacteria were susceptible to amoxicillin–clavulanate.
3	Amoxicillin–clavulanate + marbofloxacin	-	2	2 died	Two cats died in pre-diagnosed treatment.
4	Amoxicillin–clavulanate + marbofloxacin + metronidazole	-	3	3 recovered	Three cats recovered in pre-diagnosed treatment.
5	Cephalexin + metronidazole	-	1	1 died	One cat died in pre-diagnosed treatment.
6	Cephalexin + metronidazole	Switched to amoxicillin–clavulanate	1	1 died	Infection relapsed after an apparently full recovery, the owner decided to stop the treatment.
7	Cephalexin + marbofloxacin + metronidazole	-	1	1 recovered	One cat recovered in pre-diagnosed treatment.
8	Ceftriaxone + enrofloxacin + metronidazole	-	1	1 recovered	Cat recovered in pre-diagnosed treatment.
9	Enrofloxacin + metronidazole	-	1	1 died	The cat died on the day of presentation.
10	Marbofloxacin + metronidazole	-	6	2 died, 4 recovered	Two cats died in pre-diagnosed treatment. Four cats recovered in pre-diagnosed treatment.
11	Marbofloxacin + metronidazole	Remained marbofloxacin + metronidazole	1	1 recovered	Bacteria were susceptible to marbofloxacin

**Table 11:** Case outcome of the cats across different treatments or conditions.

Status (*n* = 28)	Number of Cases (*n*)	Percentage *n* (%)
Survived	13	46.4
Dead	15	53.6
Cats with chest tube	18	64.3
Survived	12	66.7
Dead	6	33.3
Cats without chest tube	10	35.7
Survived	1	10.0
Dead	9	90.0
Cats with unilateral pleural effusion	9	32.1
Survived	4	44.4
Dead	5	55.6
Cats with bilateral pleural effusion	19	67.9
Survived	9	47.4
Dead	10	52.6

**Table 12:** Complications of chest tube placement and thoracic lavage.

Complication (*n* = 11)	Number of Cases (*n*)	Percentage (%)
Lung bullae	3	27.3
Subcutaneous emphysema	3	27.3
Infection at site of chest tube placement	3	27.3
Pneumothorax	2	18.2
Failure of recovery of instilled lavage	2	18.2
Leakage of pleural fluid	1	9.1
Displaced chest tube	1	9.1

## Data Availability

The datasets used and/or analysed during the current study are available from the corresponding author upon reasonable request.

## Abbreviations

spp.	Species (plural)
BCS	Body condition score
RL	Reference limit
RBC	Red blood cell
PCV	Packed cell volume
CWCC	Complete white cell count
WBC	White blood cell
ALT	Alanine transaminase
ALP	Alkaline phosphatase
GGT	Gamma glutamyl transferase
AST	Aspartate transaminase
TP	Total protein
A:G	Albumin:globulin ratio
SE	Standard error
g	Gram
L	Litre
mmol	Millimoles
IV	Intravenous
PO	Per oral
SQ	Subcutaneous
n	Sample size
FeLV	Feline leukaemia virus
FIV	Feline immunodeficiency virus
UK	United Kingdom
PCR	Polymerase chain reaction
